# Implantation of a Self-Expanding Transcatheter Pulmonary Valve as a Salvage Procedure After Embolization of a Prestent

**DOI:** 10.1016/j.jaccas.2026.108980

**Published:** 2026-06-03

**Authors:** Christina Botrous, Dan M. Dorobantu, Mark S. Turner, Demetris Taliotis, Radwa Bedair

**Affiliations:** a Cardiology Department, Bristol Heart Institute, University Hospitals Bristol and Weston NHS Trust, Bristol, United Kingdom; b Children's Health and Exercise Research Centre (CHERC), University of Exeter Medical School, Exeter, United Kingdom; c Cardiology Department, Bristol Royal Hospital for Children, University Hospitals Bristol and Weston NHS Trust, Bristol, United Kingdom

**Keywords:** balloon-expandable valve, congenital heart disease, interventional cardiology, percutaneous valve implantation, pre-stent embolization, pulmonary regurgitation, pulmonary valve replacement, self-expanding valve, tetralogy of Fallot (TOF), transcatheter pulmonary valve implantation (TPVI), Venus-P valve

## Abstract

**Background:**

Transcatheter pulmonary valve implantation is well established for patients with repaired tetralogy of Fallot and pulmonary valve dysfunction. We report a case of a procedural complication salvaged using a self-expanding valve deployed innovatively.

**Case Summary:**

A 23-year-old male with repaired tetralogy of Fallot and severe pulmonary regurgitation underwent transcatheter pulmonary valve implantation. An Edwards Sapien 3 valve with Cheatham Platinum prestenting was planned. The procedure was complicated by Cheatham Platinum stent embolization into the cry (main pulmonary artery [MPA]), with unsuccessful repositioning. A self-expanding Venus-P valve was deployed through the embolized stent, trapping it against the MPA wall, restoring valve function, and avoiding emergency surgery.

**Discussion:**

This case highlights challenges of balloon-expandable valves in large MPA anatomy and the versatility of self-expanding valves in managing complications.

**Take-Home Messages:**

Self-expanding valves can provide effective bailout options for complications such as embolized stents. Expertise in different valve platforms allows for decision-making flexibility.

## Visual Summary

**Figure:**
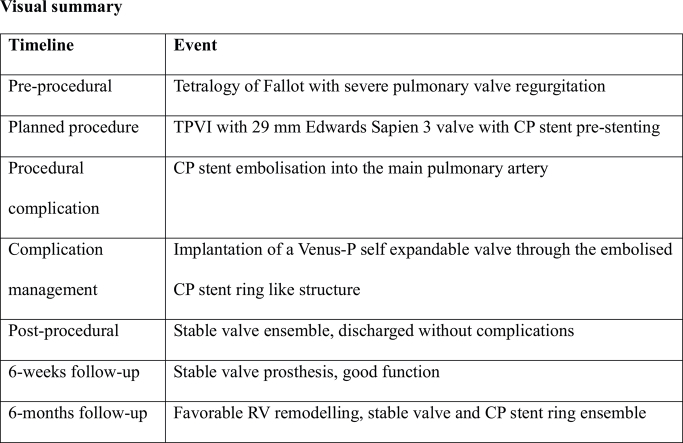

## History of presentation

**Figure 1:**
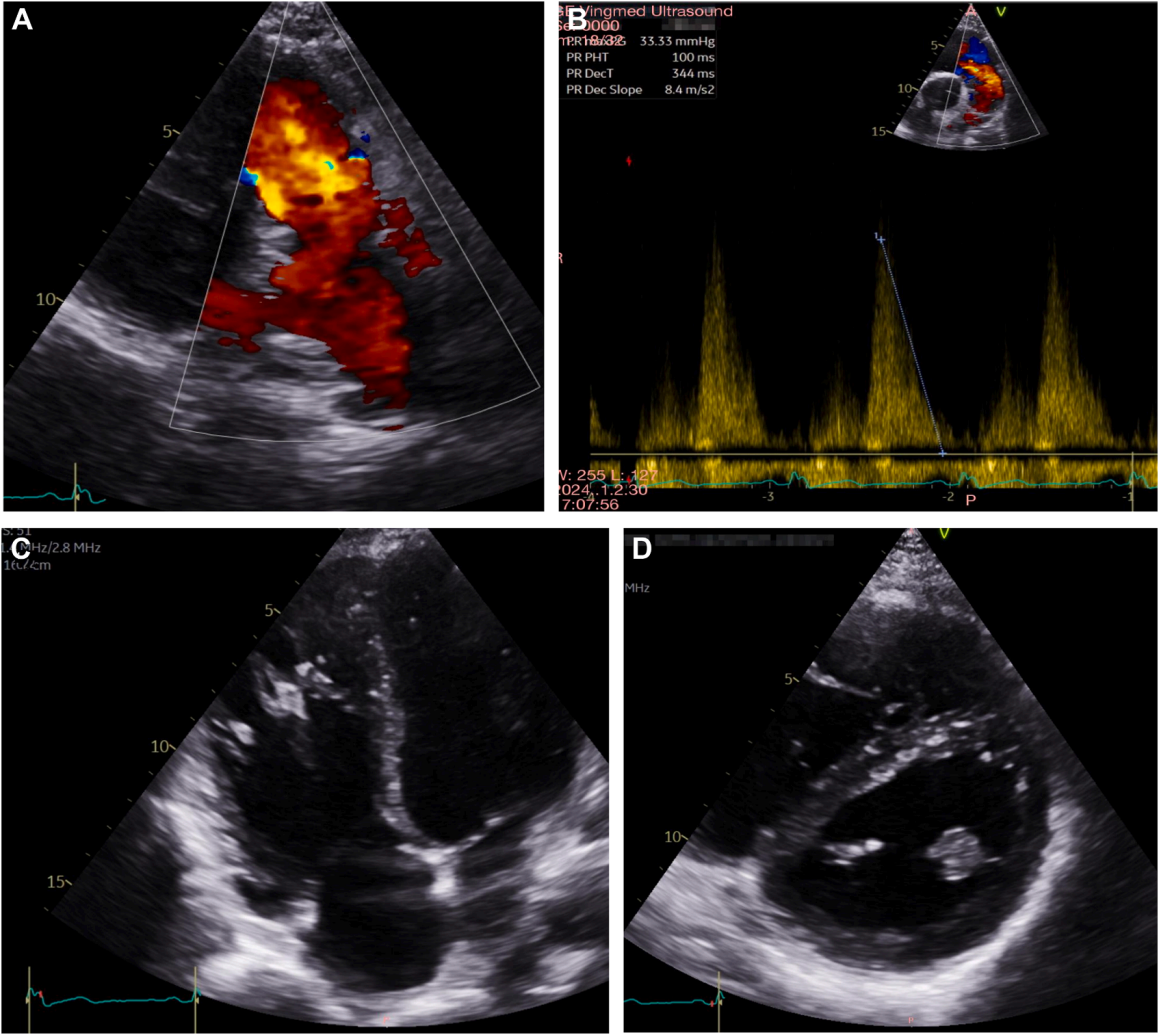
Transthoracic Echocardiogram Prior the Procedure There is free-flow pulmonary regurgitation (a); steep downslope of the continuous wave Doppler of the regurgitation, pressure half time is 100 ms (b); dilated right ventricle on 4-chamber view (c); and diastolic septal flattening on the short parasternal view (d).

A 23-year-old male with surgically repaired TOF, which included excision of the infundibular muscle band with a transannular patch, closure of ventricular septal defect, and re-construction of the main pulmonary artery (MPA) using pericardial tissue at the age of 1 year, was scheduled for TPVI for symptomatic free pulmonary regurgitation with a dilated and impaired right ventricle (RV) Figures 1A to 1D and Video 11, Video 12, Video 13.

**Take-Home Messages**

• Self-expanding transcatheter valves can offer an effective solution for complications like embolized prestents in complex pulmonary valve interventions.
• Access to and expertise in different valve platforms allow for management of complications and procedural flexibility.

## Investigations

**Figure 2:**
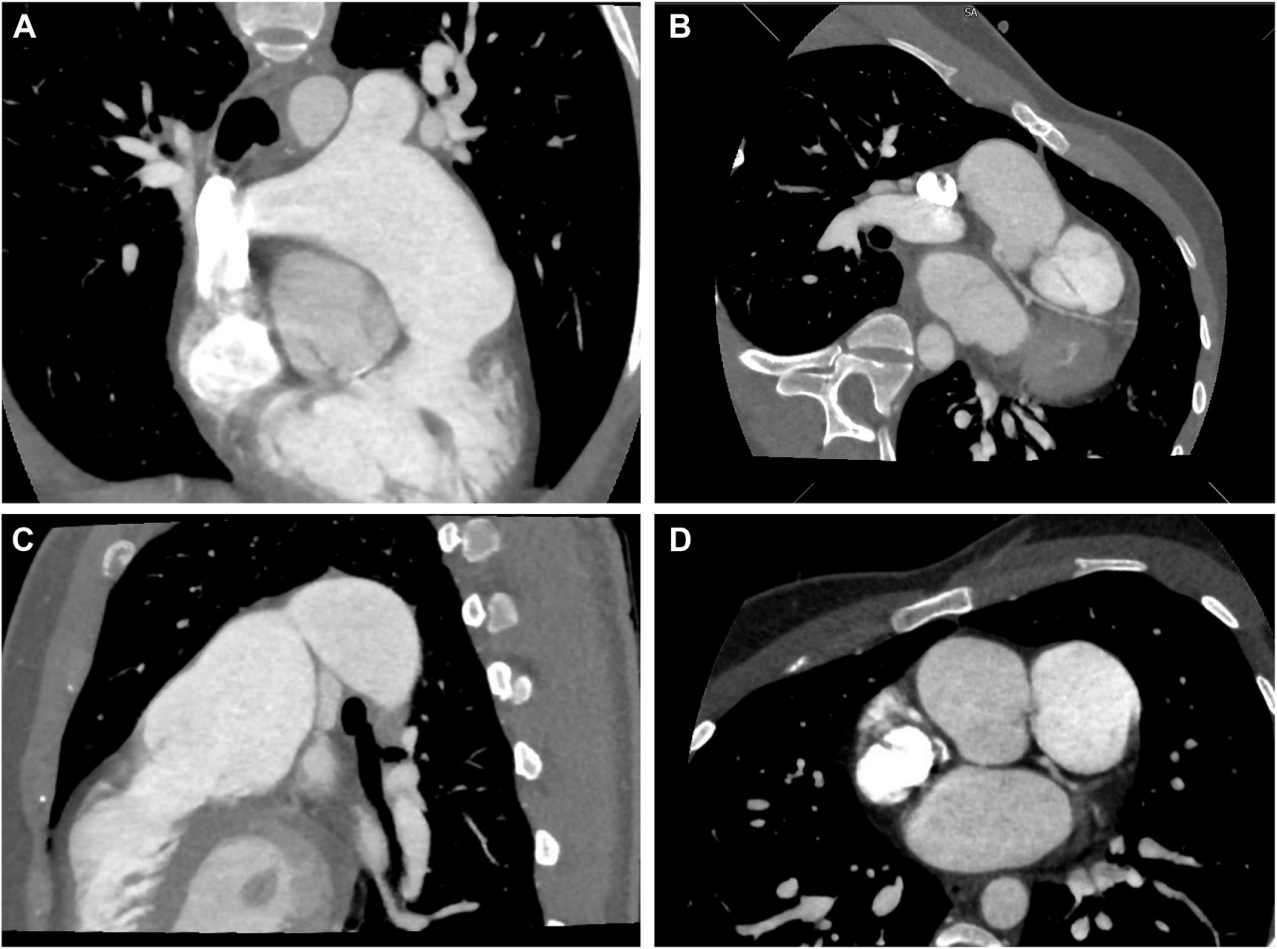
Cardiac Computed Tomography Planning Prior to the Procedure Three-dimensional construction of the RVOT and the pulmonary annulus showing different measurements in systole and the proximity of the pulmonary annulus to the left anterior descending artery (LAD) (a-e). RVOT = right ventricular outflow tract.

Cardiac magnetic resonance imaging showed severe pulmonary regurgitation (regurgitant fraction 54% to 60%) with significant RV dilatation, with a right ventricular end-diastolic volume of 239 mL (126 mL/m^2^), compared with a left ventricular end-diastolic volume of 153 mL (left ventricle 81 mL/m^2^). A gated cardiac computed tomography (CT) scan was performed for procedural planning. All CT measurements were obtained during systole, demonstrating a markedly dilated MPA: proximal MPA 50 × 41 mm, mid-MPA 47 × 42 mm, and distal MPA at the bifurcation 49 × 44 mm. A relative constriction at the annular level measured 19 × 28 mm, with a perimeter-derived diameter of 24 mm and relative proximity to the left anterior descending coronary artery at this level (Figures 2A to 2D).

With the constriction at annular level, both balloon-expanding and self-expanding valves were considered. The self-expanding valves available at our center included the Venus-P valve and the Harmony transcatheter pulmonary valve. CT-based valve simulations were performed, and both systems were deemed feasible, with the anatomy more favorable for the Venus-P valve; these simulations were based on CT imaging acquired in 2021.

## Management: Intervention

**Figure 3:**
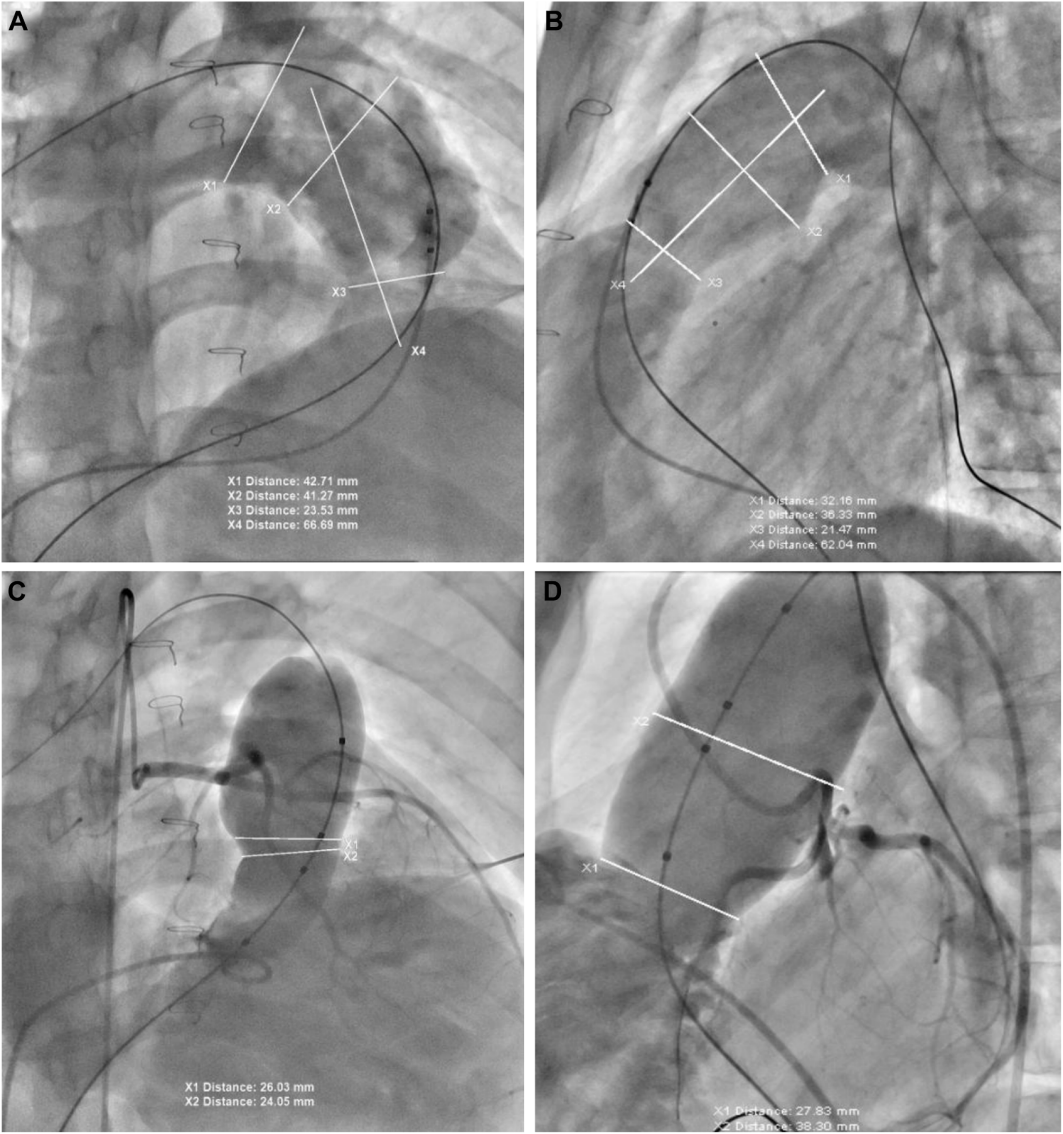
Initial Pulmonary Angiogram It shows different measurements of the pulmonary annulus and the main pulmonary artery taken in systole (a, b) and sequential main pulmonary artery and coronary angiogram (c, d).

The interventional procedure was performed under general anesthesia. Balloon interrogation using a 40 mm × 6 cm percutaneous transluminal stenting balloon (model type) (PTS-X) balloon showed an annular diameter of 27 mm, with no compromise of left coronary artery flow during simultaneous selective coronary angiography (Figures 3A to 3D).

**Figure 4:**
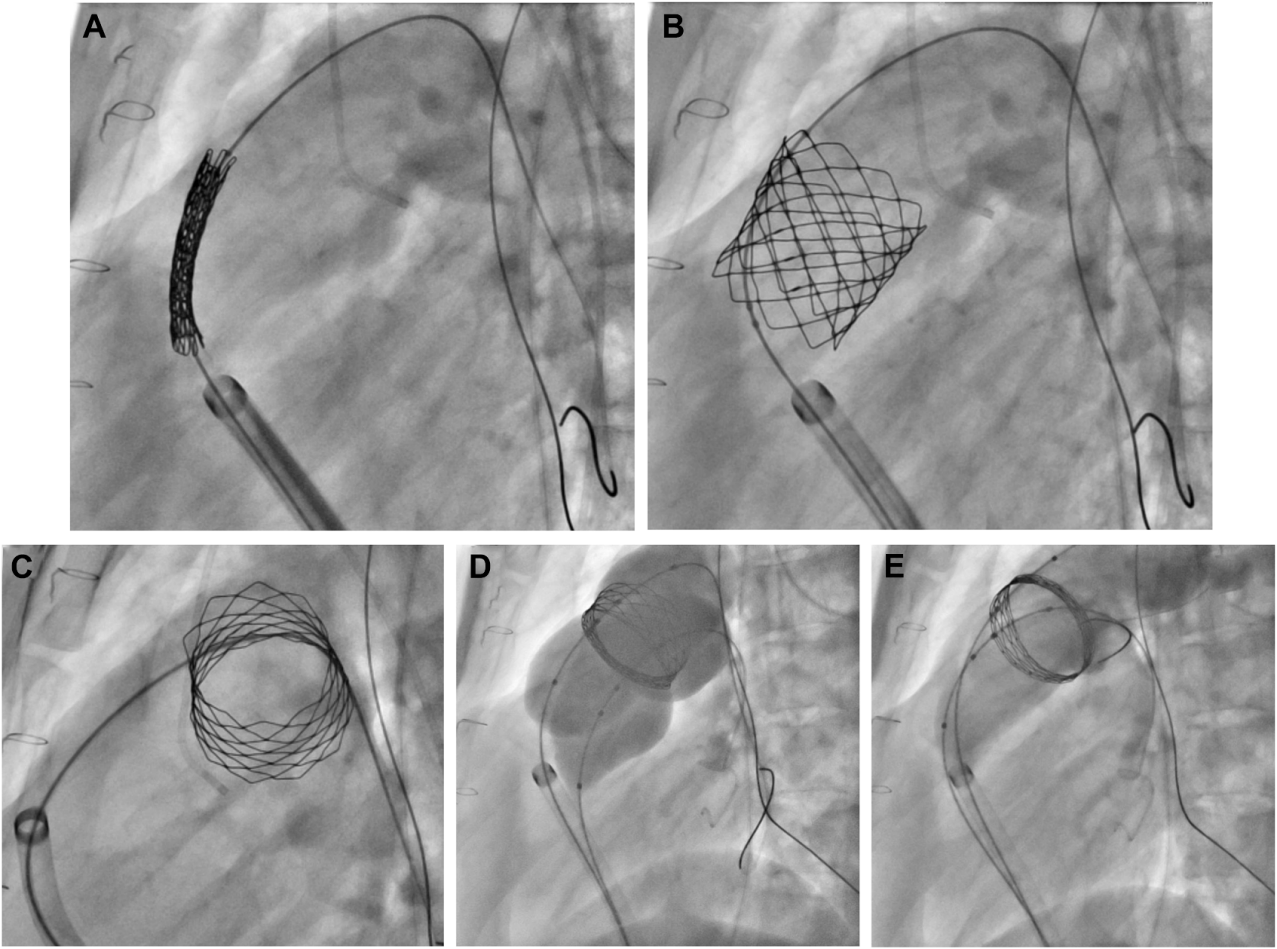
Initial Positioning of the Cheatham Platinum Stent This stent (a and b) got displaced and became unstable distally into the main pulmonary artery (MPA) (c). A simultaneous two-balloon dilation of the stent to attempt to oppose it to the MPA. Due to the aneurysmal MPA, the stent, even though expanded into a ring, was floating freely on the wire (e).

A 29-mm Edwards Sapien 3 valve with pre-stenting was chosen over a self-expanding Venus-P valve for its shorter length and perceived lower risk of ventricular dysrhythmia. A 5-cm bare 10-zig Cheatham Platinum (CP) stent was used, with the 10-zig configuration allowing potential expansion to 30 mm. The stent was crimped onto a 30-mm Balloon Inflation and Biopsy balloon and delivered via an 18F Cook sheath. The stent was advanced into position and deployed to nominal pressures. On deployment to nominal pressures, the stent shortened, particularly from the proximal end, and was inadequately positioned with only 1 zig hanging on the annulus/waist. We considered implanting a second longer stent within the stent to anchor it but were concerned that this would lead to stent embolization while advancing a sheath. We therefore elected to attempt to anchor the stent by inflating a 40 mm × 6 cm PTS-X balloon within the stent, locking it, and attempting to draw it back. This maneuver led to further stent shortening with embolization, and the stent could not be drawn back into the annulus. We then decided to expand the stent as much as possible to anchor it into the wall of the MPA and attempted this using 2 simultaneous balloons (46-mm Coda balloon and a 40 mm × 6 cm PTS-X balloon) with guidewires in the right pulmonary artery. This led to asymmetric shortening and dilatation of the stent, so it was repeated using 2 40 mm × 6 cm PTS-X balloons, and although this dilated the stent fully to resemble a ring, apposition to the wall of the MPA was still not possible given the aneurysmal size of the MPA. The stent ring measured 33 × 35 mm in diameter and was freely mobile on the wire (Figures 4A to 4E, Video 11, Video 12, Video 13 and Video 11, Video 12, Video 13).

The surgical team was contacted as we considered transfer to theatres for removal of the stent and surgical pulmonary valve replacement.

The patient was hemodynamically stable, and we therefore explored implanting a Venus-P valve beyond the “ring” of the embolized stent, locking the stent between the valve and the MPA.

**Figure 5:**
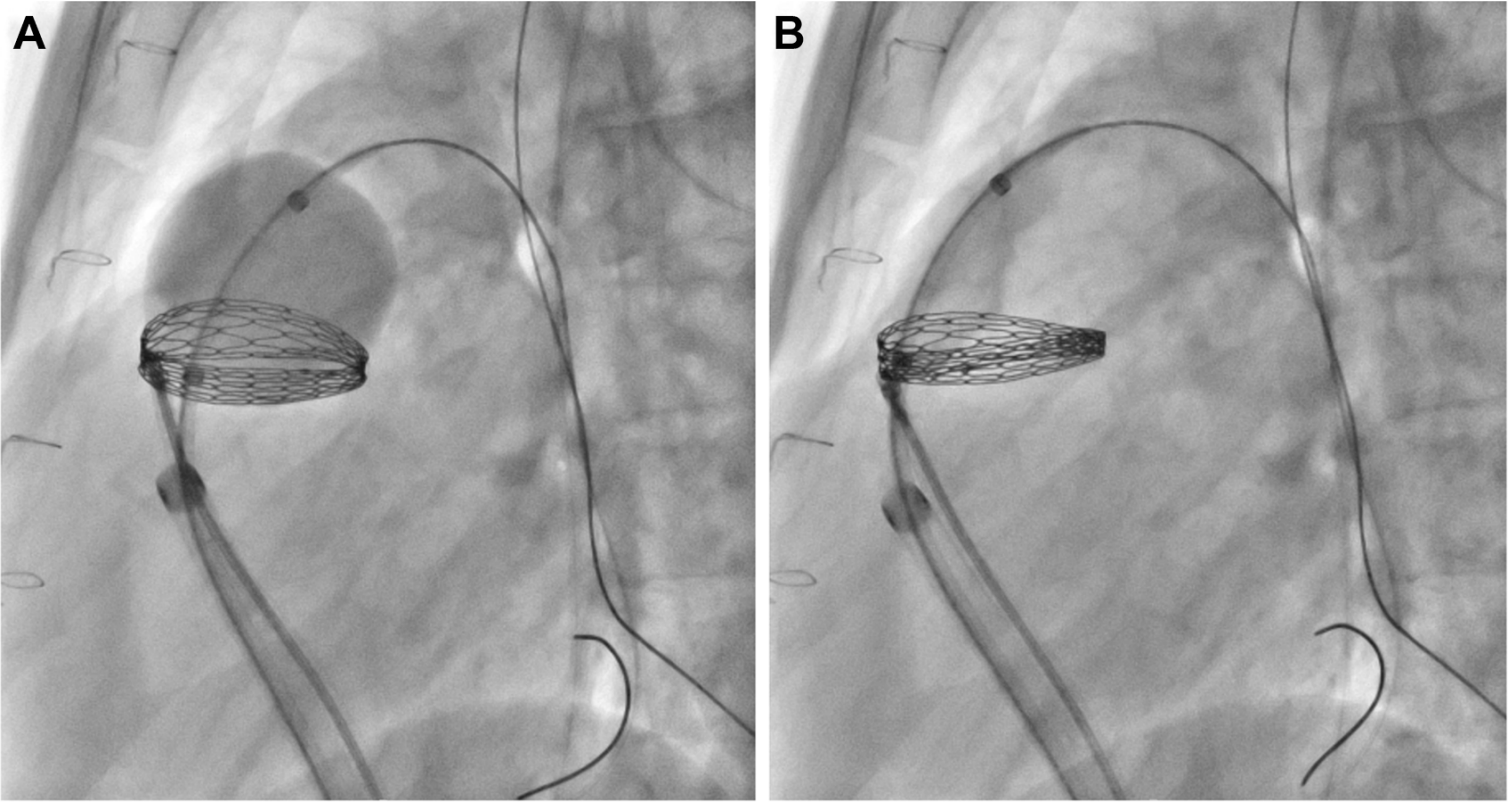
Stent Stabilization (A) The ring-like Cheatham Platinum stent was stabilized using an inflated distal Coda balloon to facilitate engagement with a Bioptome catheter. (B) Following successful capture, the Coda balloon was deflated while the Cheatham Platinum stent remained securely grasped by the Bioptome catheter.

To stabilize the embolized stent, a Coda balloon was inflated distal to the stent, facilitating capture of the proximal edge using a bioptome catheter. The stent was repositioned to the mid-MPA and stabilized in this location (Figures 5A and 5B, Video 11, Video 12, Video 13).

**Figure 6:**
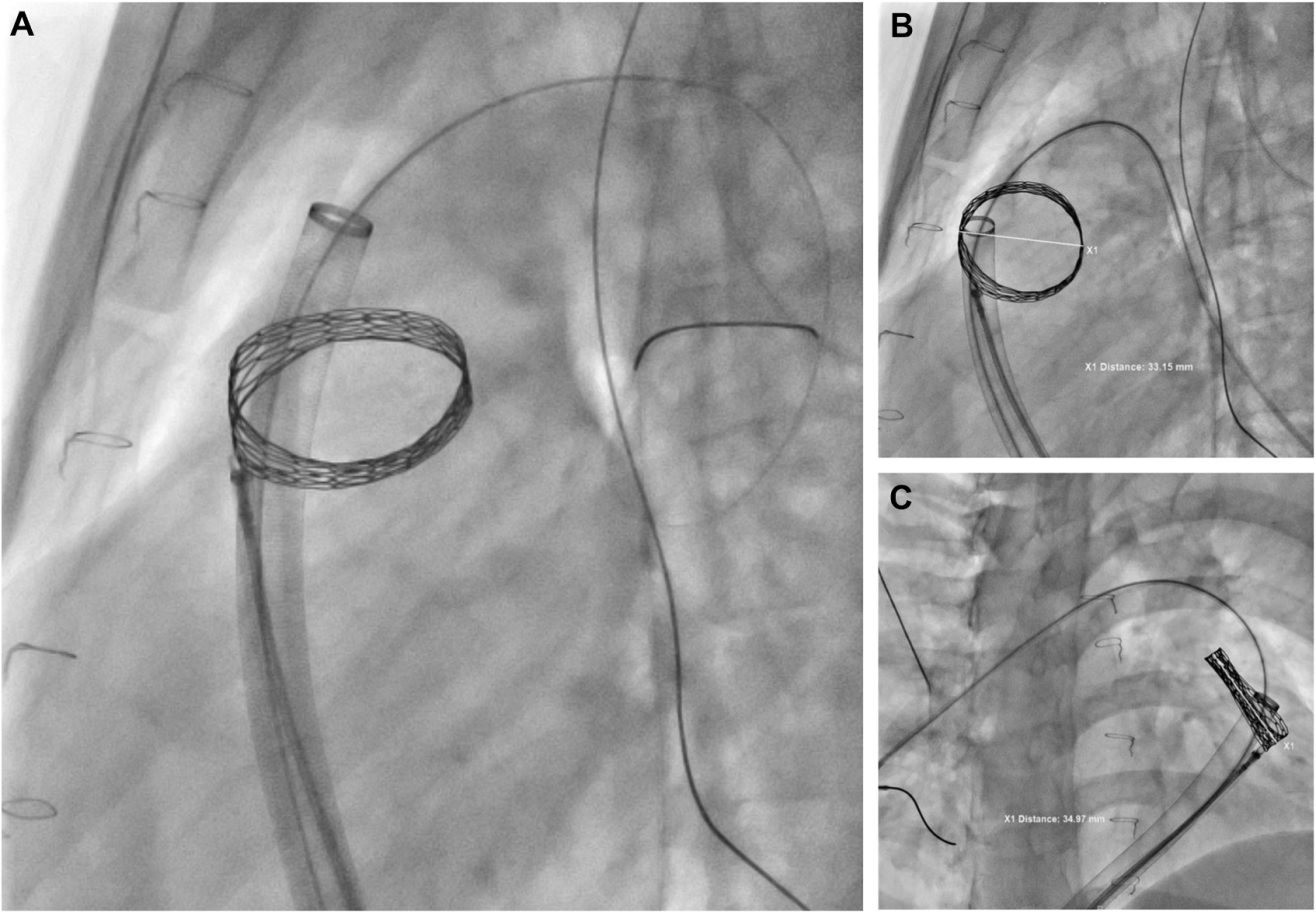
Cheatham Platinum Stent Sizing Panels show advancing of the 26F Gore DrySeal sheath into the main pulmonary artery (a) and the sizing of the ring-like Cheatham Platinum stent in both lateral (33 mm) and ROA views (35 mm) (b and c, respectively).

A 26F Gore DrySeal sheath was advanced into the MPA. A 32 × 25-mm Venus-P self-expanding valve was selected as the distal flare was 42 mm, which is larger than the measured expanded stent ring diameter of 33 × 35 mm. The Venus-P valve was advanced into the right pulmonary artery, with the bioptome maintaining the stent within the MPA (Figures 6A to 6C, Video 11, Video 12, Video 13 and Video 11, Video 12, Video 13).

**Figure 7:**
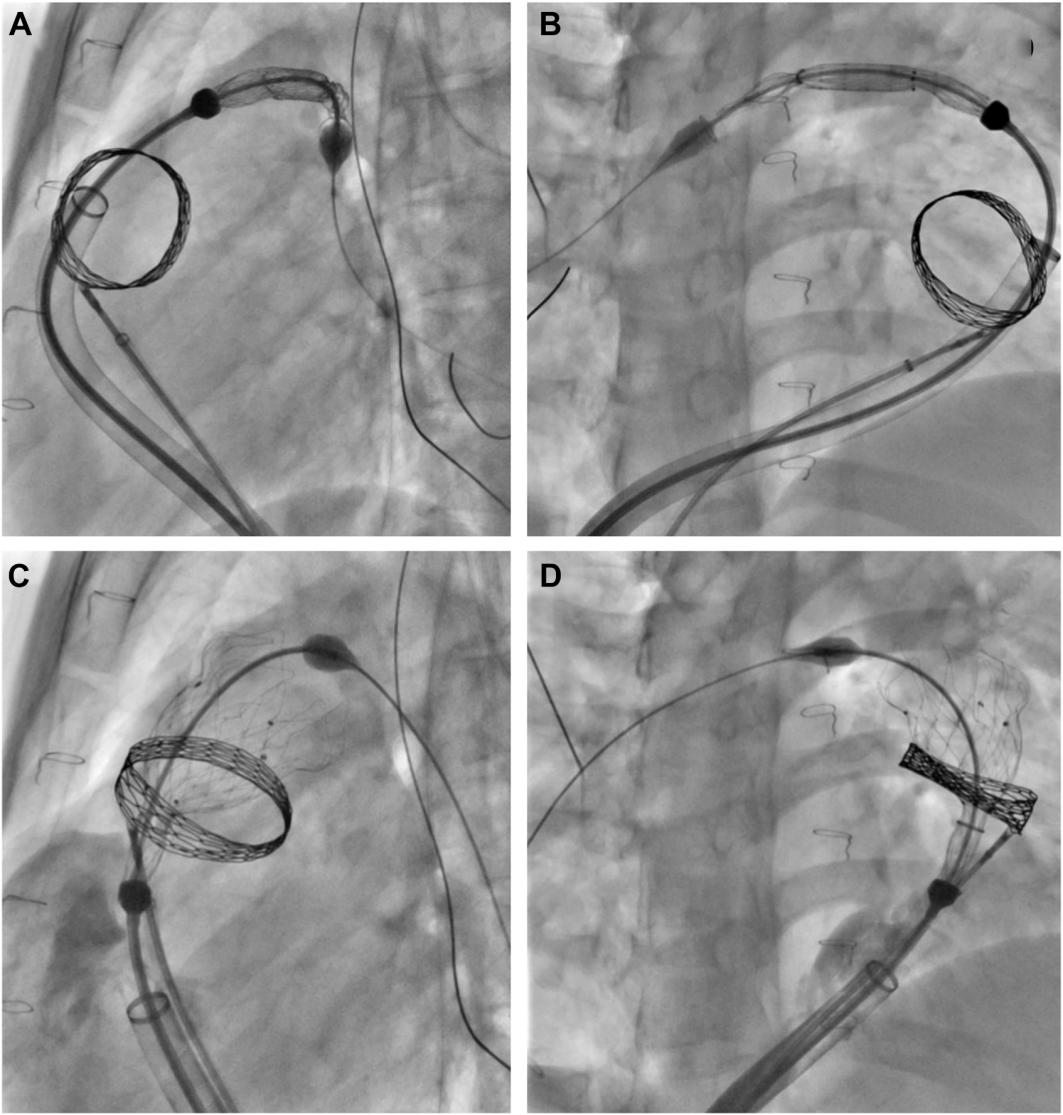
Venus-P Valve Positioning Figure shows advancing Venus-P valve distal into the main pulmonary artery (a and b are lateral and RAO views, respectively) followed by uncovering the proximal crown of the valve (c and d on same views).

**Figure 8:**
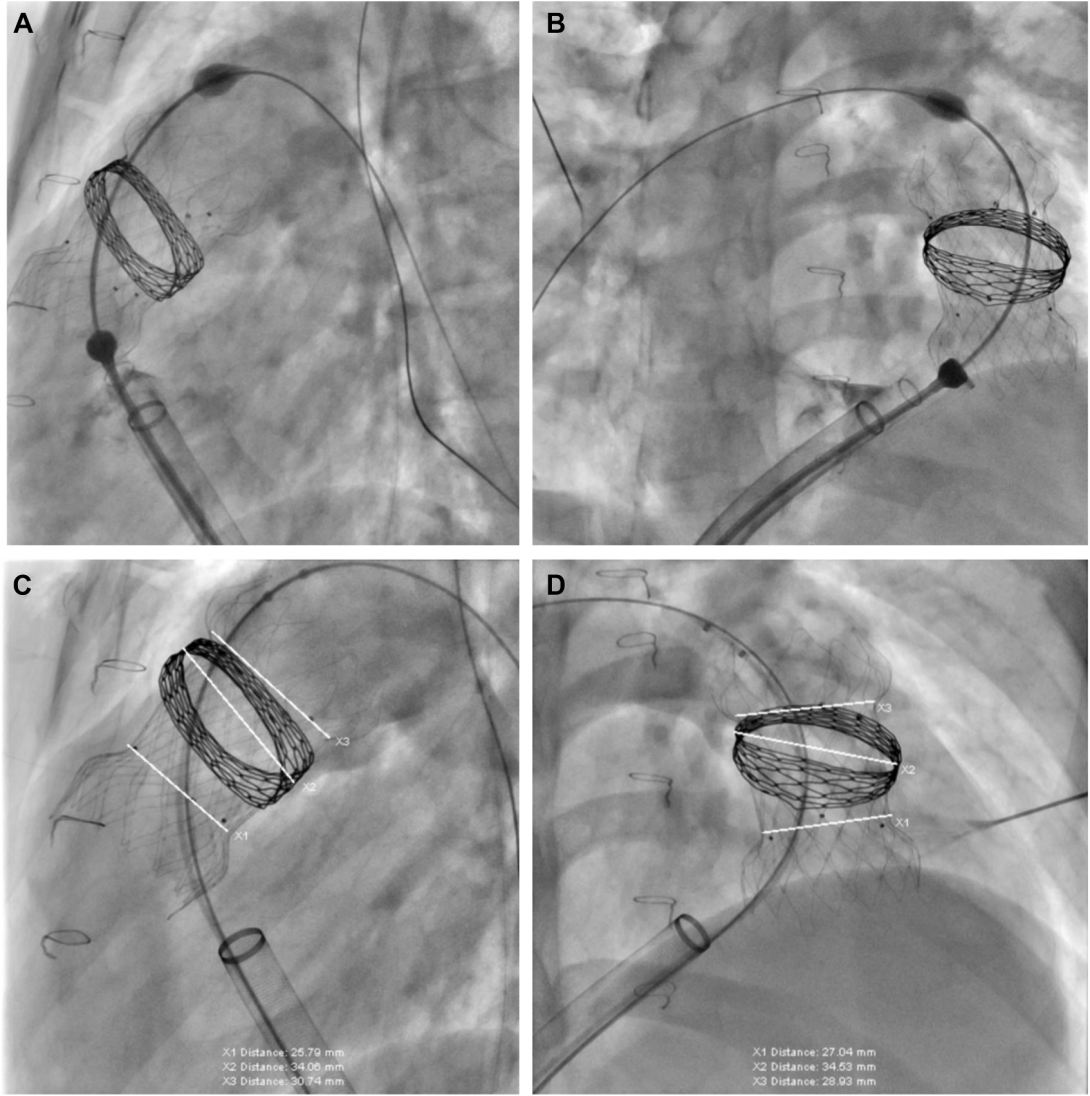
Final Uncovering of the Venus-P Valve Figure shows final uncovering step of the Venus-P valve in both lateral and RAO views (a and b, respectively) and the final measurements on the same views (c and d).

The valve was uncovered to the mid portion with appropriate flaring into the proximal PA bifurcation. While still holding onto the stent with the bioptome, the valve was retracted backwards, aiming for a transannular implant while performing angiograms through the side arm of the DrySeal sheath. The stent was now behind the distal flare. Once the straight part of the valve was in a transannular position, the bioptome was retracted, and the valve was completely unsheathed. Final angiograms showed a satisfactory position with no evidence of paravalvular leak. There was no gradient at the end procedure across the valve (Figures 7A to 7D, Figures 8A to 8D, Video 5, Video 6, Video 7, Video 8, Video 9, Video 10).

## Outcome and Follow-Up

Following the procedure, the patient remained hemodynamically stable with no ventricular arrhythmias observed during the hospital stay. A predischarge transthoracic echocardiogram confirmed a stable valve position and good valve function.

**Figure 9:**
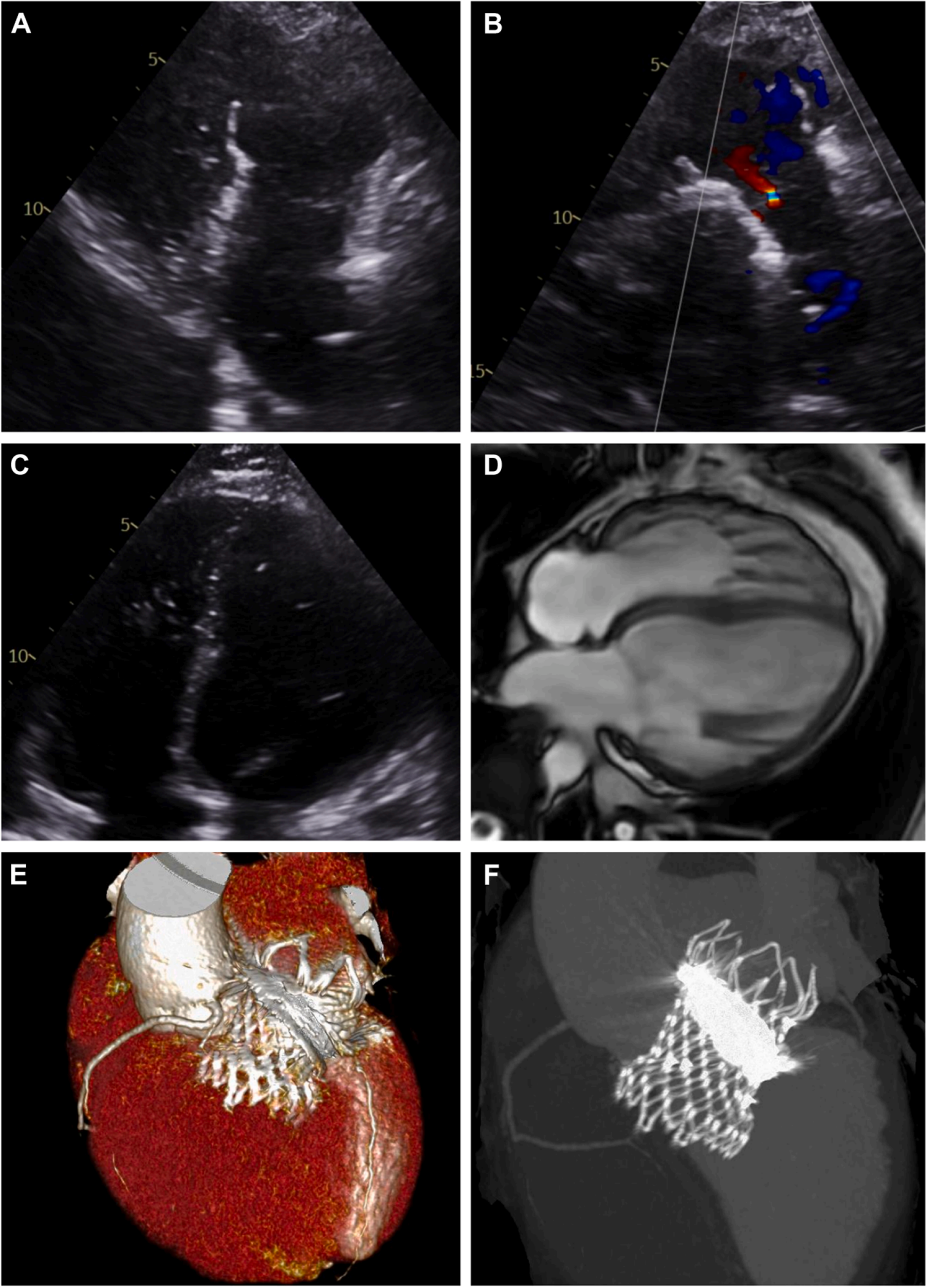
Follow-Up Imaging There were good results on the transthoracic echocardiogram at 3 months postprocedure with good position of the Venus-P valve in the modified RVOT view (a) and only minimal regurgitations demonstrated on colour flow and Pulsed wave Doppler (b) with favorable RV size reduction at 3 months by echocardiogram (c) and at 6 months by cardiac MRI (d). The CT obtained at 6 months shows the ring-like Cheatham Platinum stent and a Venus-P valve ensemble stable in the RVOT and main pulmonary artery in relation to the right and left coronary arteries (e-f). CT = computed tomography; MRI = magnetic resonance imaging; RV = right ventricle; RVOT = right ventricular outflow tract.

At 6-week follow-up, the patient reported marked symptomatic improvement in breathlessness. Repeat echocardiography demonstrated a stable valve position with good valve function and only trivial pulmonary regurgitation, as well as a reduction in right ventricular size. At 6 months, the repeat cardiac MRI showed favorable RV size remodeling from an end-diastolic volume of 239 mL preprocedurally to 163 mL after (Figures 9A to 9D, Videos 14 and 15). The CT at 6 months highlights the ring-like CP stent and Venus-P valve ensemble inside the right ventricular outflow tract (RVOT) and MPA (Figure 9E, Videos 16 and 17).

## Discussion

This case represents, to our knowledge, the first report of successful salvage of an embolized pulmonary prestent using a self-expanding transcatheter pulmonary valve by trapping the stent between the valve frame and the MPA.

### Prestenting

Balloon-expandable valve systems require the presence or creation of a stable landing zone, often necessitating prestenting. In markedly dilated or aneurysmal MPA morphologies, this requirement poses technical challenges, including stent shortening, instability, and migration. Shortening of CP stents during balloon expansion is a recognized phenomenon and may result in an unpredictable movement, particularly when deployed at larger MPA diameters. This can be minimized by careful selection of stent length and meticulous positioning with anticipation of shortening behavior.^1^, ^2^, ^3^, ^4^, ^5^ In retrospect, the use of a longer (6 cm) CP stent or positioning more proximally may have reduced the risk of embolization. However, predicting symmetrical shortening remains challenging, particularly in large pulmonary arteries.

Simultaneous delivery of a prestent and balloon-expandable valve is a reported technique for TPVI. Potential advantages include improved axial stability, while disadvantages include reduced flexibility, increased delivery profile, and limited repositionability, particularly relevant in this patient's anatomy.^5^^,^^6^ It was felt that achieving a good landing zone with the prestent first would be safer in our case.

### Choice between a balloon-expandable and a self-expanding valve

Our initial decision to implant a balloon-expandable valve was driven by the excellent long-term data available for the Sapien S3 valve in the pulmonary position but also due to concerns around the risk of ventricular arrhythmia with a transannular implant of a self-expanding valve. Considering the growing experience with self-expanding valve implantation, the arrhythmia risk, which can commonly be seen acutely at the time of implant, is less of a long-term concern, particularly in a TOF cohort of patients.^7^, ^8^, ^9^, ^10^

Although balloon-expandable valves have been used extensively for native-outflow RVOT morphologies, the valve design does not conform to the morphology in the same way that the new self-expanding valve platforms can. Self-expanding valves are designed for the complex native RVOT, and correct positioning is undoubtedly less technically challenging.^7^

In this case, although the MPA dimensions exceeded the recommended sizing range for supra-valvular implantation of self-expanding valves available in the UK, the presence of an annular-level restriction was felt to allow for a safe transannular implantation strategy.

## Conclusions

This case highlights the importance of procedural adaptability in the context of complications and/or new findings. The immediate access to multiple valve platforms is desirable. Comprehensive preprocedural imaging and planning remains critical, but the ability to modify strategy in response to unexpected complications is equally essential to achieving optimal outcomes.

## Funding Support and Author Disclosures

This work was supported through an unrestricted educational grant awarded to University Hospitals Bristol & Weston NHS Foundation Trust by Venus Medtech (Hangzhou) Inc (grant number: cc 105065). The authors have reported that they have no relationships relevant to the contents of this paper to disclose.

## Appendix

Video 1 Three Steps of Initial Prestent Positioning Followed by Balloon Inflation and Subsequent Embolization

Video 2 Attempt to Prepare the Embolized Stent to Implant a Second Longer Stent Failed as Stent Had Shortened

Video 3 Holding the Now Ring-Like Stent via Bioptome Catheter After Stabilizing It Through a Distal Balloon

Video 4 Advancing 26F Gore DrySeal Sheath While Holding the Stent in Place Using the Bioptome Catheter

Video 5 Positioning the Valve Into the Distal Main Pulmonary Artery Through the Now Ring-Like Stent in Both Lateral and Right Anterior Oblique Views

Video 6 Unsheathing the Distal Flare (Outflow End) of the Venus-P Valve in Both Lateral and Right Anterior Oblique Views

Video 7 Unsheathing the Proximal End (Inflow End) of the Venus-P Valve in Both Lateral and Right Anterior Oblique Views

Video 8 Releasing the Bioptome Catheter After Full Uncovering of the Venus-P Valve

Video 9 Fluoroscopy of the Venus-P Valve in Both Lateral and Right Anterior Oblique Views

Video 10 Final Lateral and Right Anterior Oblique Views of the Venus-P Valve

Video 11 Preprocedural Right Ventricular Outflow Tract Echocardiogram

Video 12 Preprocedural Right Ventricular Function A4C Echocardiogram

Video 13 Preprocedural Right Ventricular Function SAX Echocardiogram

Video 14 Postprocedural Right Ventricular Outflow Tract Echocardiogram

Video 15 Postprocedural Right Ventricular Function A4C Echocardiogram

Video 16 Tissue 3D CT Rotational Reconstruction of the Heart Showing the Ring-Like CP Stent and Venus-P Valve Ensemble Stable in the Right Ventricular Outflow Tract and MPA and Coronary Arteries

Video 17 Three-dimensional computed tomography maximum intensity projection Rotational Reconstruction of the Heart Showing the Ring-Like CP Stent and Venus-P Valve Ensemble Stable in the Right Ventricular Outflow Tract and MPA MIP 3D CT Rotational Reconstruction of the Heart Showing the Ring-Like CP Stent and Venus-P Valve Ensemble Stable in the Right Ventricular Outflow Tract and MPA
